# Estimation of disability weight for paragonimiasis: a systematic analysis

**DOI:** 10.1186/s40249-018-0485-5

**Published:** 2018-10-19

**Authors:** Yun Feng, Thomas Fürst, Lu Liu, Guo-Jing Yang

**Affiliations:** 1 Jiangsu Institute of Parasitic Diseases, 117 Meiyuan Yangxiang, Wuxi City, Jiangsu Province 214064 People’s Republic of China; 2 Key Laboratory of National Health and Family Planning Commission on Parasitic Disease Control and Prevention, Wuxi, People’s Republic of China; 3 Jiangsu Provincial Key Laboratory on Parasite and Vector Control Technology, Wuxi, People’s Republic of China; 4 Department of Epidemiology and Public Health, Swiss Tropical and Public Health Institute, Socinstrasse 57, CH-4002 Basel, Switzerland; 5 University of Basel, Basel, Switzerland

**Keywords:** Paragonimiasis, Disability weight, Disease burden, Disability-adjusted life years

## Abstract

**Background:**

Paragonimiasis, caused by helminths of the genus *Paragonimus* spp., is a neglected tropical disease. Human suffering from paragonimiasis is often misunderstood and its quantification by the disability weight of the disability-adjusted life years largely varies in different global burden of disease (GBD) estimates. This paper is to systematically review clinical paragonimiasis cases and requantify the disability weight of human paragonimiasis.

**Methods:**

A systematic analysis was conducted using articles from the following databases: PubMed, Institute for Scientific Information Web of Science, China National Knowledge Infrastructure, the Chinese scientific journal databases Wanfang Data and CQVIP, Africa Journal Online, and the System for Information on Grey Literature in Europe. Search terms were the combination of “paragonim*” with “clinical” or “infection”. Only articles fulfilling the following conditions were recruited for this study: the occurrence of clinical signs and symptoms of paragonimiasis in human beings were reported; diagnosis was confirmed; no comorbidities were reported; the reviewed clinical cases or epidemiological findings were not already included in any other articles. The information and frequencies of paragonimiasis outcomes from included articles using predefined data fields were extracted two times by two separate individuals. Outcome disability weights were selected mainly from the GBD 2004 and GBD 2013 datasets. Frequencies and disability weights of paragonimiasis outcomes were modelled into a decision tree using the additive approach and multiplicative approach, respectively. Monte Carlo simulations were run 5000 times for an uncertainty analysis.

**Results:**

The disability weight estimates of paragonimiasis were simulated with 5302 clinical cases from 80 general articles. The overall disability weight was estimated at 0.1927 (median 0.1956) with a 95% uncertainty interval (UI) of 0.1632–0.2378 using the additive approach, and 0.1791 (median 0.1816) with a 95% UI of 0.1530–0.2182 using the multiplicative approach. The simulated disability weights of *Paragonimus westermani* cases were higher than that of *P. skrjabini* cases. Lung outcomes and headache were the top two contributors to disability weight for both species.

**Conclusions:**

The use of paragonimiasis disability weight needs to be reconsidered with regard to availability of morbidity data and species variation. Calculating the disease burden of paragonimiasis requires further modification and thus has considerable implications for public health prioritization in research, monitoring, and control.

**Electronic supplementary material:**

The online version of this article (10.1186/s40249-018-0485-5) contains supplementary material, which is available to authorized users.

## Multilingual Abstracts

Please see Additional file 1 for translations of the abstract into the five official languages of the United Nations.

## Background

Paragonimiasis is a neglected tropical disease (NTD) [1] caused by helminths of the genus *Paragonimus* spp., whose eggs hatch in freshwater and yield motile miracidia. The miracidia further develop into motile microcercous cercariae in freshwater snails and later grow into metacercariae in crustaceans like crab or crayfish. Ingestion of contaminated, undercooked freshwater crabs or crayfish leads to paragonimiasis [2].

Paragonimiasis is not usually considered a national or regional public health priority, but due to its wide distribution and severe health consequences in endemic regions, the disease should not be neglected. Paragonimiasis is endemic in parts of Africa, North and South America, and particularly in Southeast and East Asian countries such as China, Japan, and the Philippines [3, 4]. The prevalence of paragonimiasis in northeastern India can reach as high as 51.7% in children aged under 15 years and 18.7% in adults [5]. The disease can also occur in non-endemic areas; for instance, in returning travellers or through infections caused by internationally traded and contaminated raw freshwater crab or crayfish [2, 6]. With the gradually increasing aquaculture production of freshwater crustacea worldwide [7], the endemicity and re-emergence of paragonimiasis deserve more attention [8].

Paragonimiasis causes complex symptoms in multiple organs. Chest symptoms are its most remarkable clinical features, as it is mainly in the lung parenchyma that adult worms exist in pairs encapsulated in fibrous cysts [2, 9]. For some *Paragonimus* species such as *P. skrjabini*, only a few of the parasites develop into adults in the lungs, while most others migrate to various organs including muscles, subcutaneous tissue, brain, liver, and so on, at the juvenile stage [9, 10]. Such ectopic infections can cause a variety of signs and symptoms including subcutaneous mass, headache, epilepsy, diarrhoea [9, 11, 12].

Probably due to its status as a NTD, suffering from paragonimiasis is often misunderstood and its quantification in the disability weight (DW) of disability-adjusted life years (DALYs) largely varies in global burden of disease (GBD) estimates. Fürst et al. [3] estimated the disease burden of paragonimiasis based on existing DWs pertaining to most similar outcomes, which were chronic outcomes of lower respiratory infection for heavy pleuropulmonary infections and seizure disorders due to meningitis for cerebral paragonimiasis (see also [13]). The GBD 2010 study used the newly developed DW of the health status “moderate abdominal pelvic problem” for the outcomes of both heavy pleuropulmonary and cerebral paragonimiasis [14, 15]. As it is clinically incorrect, and signs and symptoms of pleuropulmonary and cerebral paragonimiasis do not really match those of moderate abdominopelvic problems, the GBD 2013 used the DWs for tuberculosis (TB) and epilepsy as proxies [16, 17]. The GBD 2016 continued with similar usage, but an extra proxy DWs of chronic obstructive pulmonary disease (COPD) with different severity levels were also used [18] (see Table 1). In fact, cases of pleuropulmonary paragonimiasis often present with asthma-, bronchitis-, and TB-like signs and symptoms, but their general health state is usually better than that of TB patients [9, 11, 19]. Realizing this and relying on the then most recent DWs from the GBD 2010 study, the World Health Organization’s Foodborne Disease Burden Epidemiology Reference Group (WHO FERG) applied the DW for uncontrolled asthma instead of the more than two times higher DW for TB as a proxy for pleuropulmonary paragonimiasis in their latest estimates [15, 20, 21].

**Table 1:** DWs used in disease burden estimation

Disease burden estimation	Outcome	Proxy health state	Proxy DW	Reference
GBD 2016	Asymptomatic paragonimiasis	N/A	N/A	[18]
	Heavy paragonimiasis	TB, not HIV infected	0.333 (0.224–0.454)	
	Mild paragonimiasis due to foodborne trematodiases	COPD and other chronic respiratory problem, mild	0.019 (0.011–0.033)	
	Moderate paragonimiasis due to foodborne trematodiases	COPD and other chronic respiratory problem, moderate	0.225 (0.153–0.31)	
	Severe paragonimiasis due to foodborne trematodiases	COPD and other chronic respiratory problem, severe	0.408 (0.273–0.556)	
	Cerebral paragonimiasis	Epilepsy, less severe (seizure < 1 per month)	0.263 (0.173–0.367)	
		Epilepsy, severe (seizure ≥1 per month)	0.552 (0.375–0.710)	
GBD 2013	Asymptomatic paragonimiasis	N/A	N/A	[16, 17]
	Heavy paragonimiasis	TB, not HIV infected	0.333 (0.224–0.454)	
	Cerebral paragonimiasis	Epilepsy, less severe (seizure < 1 per month)	0.263 (0.173–0.367)	
		Epilepsy, severe (seizure ≥1 per month)	0.552 (0.375–0.710)	
WHO FERG	Paragonimiasis	Uncontrolled asthma	0.132 (0.087–0.190)	[15, 21, 22]
GBD 2010	Heavy paragonimiasis	Abdominopelvic problem: moderate	0.123 (0.083–0.176)	[14, 15, 23, 24]
	Cerebral paragonimiasis	Abdominopelvic problem: moderate	0.123 (0.083–0.176)	
Fürst et al.	Heavy paragonimiasis	Lower respiratory infection	0.099	[3]
	Cerebral paragonimiasis	Seizure disorders due to meningitis	0.100	

*DW* Disability weight, *COPD* Chronic obstructive pulmonary disease, *N/A* Not applicable, *GBD* Global burden of disease, *WHO FERG* World Health Organization Foodborne Disease Burden Epidemiology Reference Group, *TB* Tuberculosis, *HIV* Human immunodeficiency virus

The substantial differences in DWs for paragonimiasis translate into substantial differences in the thus far published GBD estimates and thereby limit the usefulness of all these estimates [3, 20, 22–25]. Also of note is that all the aforementioned estimates considered only pleuropulmonary and cerebral paragonimiasis, however, additional outcomes that were associated with paragonimiasis, such as pericarditis due to heart involvement, for instance [12], should also be considered.

Due to all these factors, we systematically reviewed clinical paragonimiasis cases and applied decision tree modelling approaches to requantify the DW for human paragonimiasis.

## Methods

### Search strategy and selection criteria

The systematic literature search included the databases PubMed, Institute for Scientific Information (ISI) Web of Science, China National Knowledge Infrastructure (CNKI), the Chinese scientific journal databases Wanfang Data and CQVIP, Africa Journal Online (AJOL), and the System for Information on Grey Literature in Europe (SIGLE). Search terms were the combination of “paragonim*” with “clinical” or “infection”. Articles were reviewed by title and abstract, and subsequently full text.

**Fig. 1:**
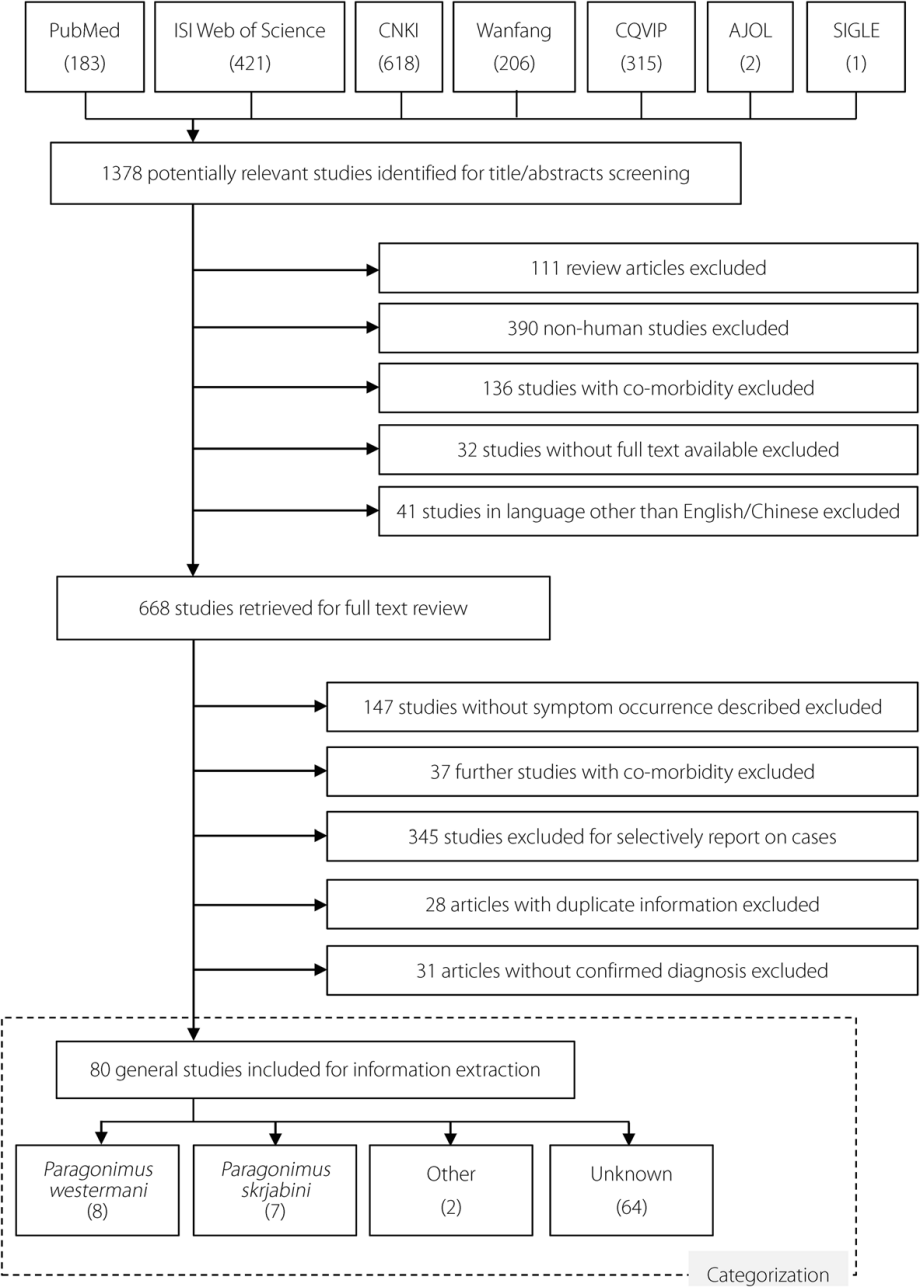
Article selection flow chart. Figure 1 provides an illustration of the process of how articles were recruited

The inclusion criteria were: (i) the articles described the occurrence of clinical signs and symptoms of paragonimiasis in human beings; (ii) the articles reported on paragonimiasis only (i.e. articles reporting obvious comorbidities were excluded); (iii) the diagnosis of paragonimiasis cases was confirmed by parasitological, serological, or histological evidence; and (iv) the article reviewed clinical cases or epidemiological findings that were not already included in other articles. The year of the study and the year of publication were irrelevant in the inclusion/exclusion of articles. Articles in languages other than English or Chinese, or without full text available to authors, were excluded. Articles that selectively reported on a specific case group such as cerebral paragonimiasis were also excluded. The study selection process is shown in Fig. 1.

### Data extraction and processing

Two authors extracted the information from included articles. Information was extracted according to the following predefined data fields: case recruitment method (case reviews/surveys); region (country, province); *Paragonimus* species; and outcomes reported and their relevant occurrences.

The reported outcomes covered the following items: lung outcome (chest X-ray/ computed tomography shadow with symptoms such as fever or cough); pleural outcome (pleural effusion with symptoms such as chest pain); pericardial outcome (pericardial effusion with symptoms such as fever or dyspnea); abdominal pain; hepatomegaly; skin rash; subcutaneous mass; headache; epilepsy; vision impairment; motor loss; and diarrhea. The frequency of each outcome was estimated by its relative occurrence in the respective group.

Another pooling was made with regard to the exact *Paragonimus* species. For Chinese articles that did not report detailed species information, the exact species was assigned according to the geographic location of the cases, if applicable. Following this rationale, which is based on the authors’ experience about the distribution of paragonimiasis species in China, cases from the Chinese provinces/municipalities Zhejiang, Shanghai, Jiangsu, Anhui, Jiangxi, Fujian, Jilin, Liaoning, and Beijing were classified as *P. westermani*, whereas cases from the provinces Shaanxi, Sichuan, Hubei, Chongqing, Guilin, and Henan were classified as *P. skrjabini*. Cases from countries such as Japan and the US remained in the ‘not further specified’ group.

Disability weights for outcomes (see Table 2) were retrieved in the following order of priority: from the GBD study, i.e. GBD 2013 [16], GBD 2004 [13], and finally – if not available from the previous sources – from other literature.

**Table 2:** DWs for various outcomes

Outcome	DW mean	DW uncertainty level	Source item	Reference
Lung outcomes	0.279	–	Lower respiratory infection episodes	GBD 2004
Pleural outcomes	0.054	0.035–0.079	Lower back pain, moderate (proxy for chest pain)	GBD 2013
Pericardial outcomes	0.252	–	Inflammatory heart disease	GBD 2004
Headache	0.441	0.294–0.588	Headache, migraine	GBD 2013
Epilepsy	0.552	0.375–0.710	Epilepsy, severe	GBD 2013
Motor loss	0.061	0.040–0.089	Motor impairment, moderate	GBD 2013
Vision impairment	0.031	0.019–0.049	Distance vision impairment, moderate	GBD 2013
Diarrhoea	0.074	0.049–0.104	Diarrhea, mild	GBD 2013
Abdominal pain	0.060	–	Abdominal pain	Finkelstein et al. 2007 [26]
Hepatomegaly	0.060	–	Mild/moderate hepatomegaly	Finkelstein et al. 2007 [26]
Skin rash	0.068	–	Oncocerciasis itching	GBD 2004
Subcutaneous mass	0.023	–	Cutaneous leishmaniasis	GBD 2004

*DW* Disability weight, *GBD* Global burden of disease

### Decision tree modelling

#### Decision tree

A decision tree was used to systematically combine the large set of outcomes and their associated DWs with the corresponding frequencies to ultimately estimate summary DWs. The additive approach (Formula 1) was better in combining outcomes that presented separately in individual patients, while the multiplicative approach (Formula 2) was more suitable for combining several outcomes occurring in one patient [27]. As both conditions existed, the two approaches were used respectively in the analysis to compare with each other. P_outcome A_ represents the probability/frequency of outcome A; DW_outcome A_ represents the DW of outcome A. The total number of outcomes considered is represented by *n* in Formulas 1 and 2. All calculations were processed using R software version 3.1.1 (the R Foundation for Statistical Computing, Vienna, Austria).

1 $$ \mathrm{DW}={\sum}_{A=1}^n{P}_{outcome\;A}\ast {DW}_{outcome\;A} $$

2 $$ \mathrm{DW}=1-{\sum}_{A=1}^n\left(1-{P}_{outcome\;A}\ast {DW}_{outcome\;A}\right) $$

### Base case analysis

First, we calculated the base case DW for each group. Second, to assess the contribution of each outcome to the overall DW, we recalculated the DW for each group supposing the specific outcome is missing. If the DW changed by more than 10%, the outcome was considered critical.

### Uncertainty analysis

For each group, an uncertainty analysis was conducted to explore the range of overall DWs when all formula inputs varied simultaneously. In each simulation, each formula input (P_outcome A_ or DW_outcome A_) was selected randomly from its specific distribution. The distribution of probability/frequencies followed a beta distribution estimated using the cases in each group. The distribution of DWs followed a logit-normal distribution [28] estimated from the reported mean and range. In total, 5000 Monte Carlo simulations were performed. After summarizing all simulations, we demonstrated the uncertainty interval (UI) by the 2.5 to 97.5% percentile range of DWs. (For details, see the Additional file 2).

## Results

Among the 80 general articles that were included in this study, 64 did not investigate the species of paragonimiasis. After estimating using cases’ locations, 38 articles were categorized in the species group of *P. westermani* and another 38 in the group of *P. skrjabini* (see Fig. 1 and Table 3).

**Table 3:** Overall paragonimiasis DW and DWs by species

Type	No. of articles	No. of cases	Additive approach		Multiplicative approach	
			DW	Uncertainty analysis [median (2.5–97.5%)]	DW	Uncertainty analysis [median (2.5–97.5%]
Overall	80	5302	0.1927	0.1956 (0.1632–0.2378)	0.1791	0.1816 (0.1530–0.2182)
Species reported^a^						
*P. westermani*	8	289	0.2829	0.2901 (0.2274–0.3791)	0.2601	0.2660 (0.2121–0.3391)
*P. skrjabini*	7	701	0.0827	0.0860 (0.0676–0.1115)	0.0804	0.0836 (0.0659–0.1078)
Other	2	130	0.2073	0.2212 (0.1646–0.2983)	0.1951	0.2067 (0.1563–0.2729)
Unknown	64	4182	0.2044	0.2077 (0.1745–0.2493)	0.1889	0.1917 (0.1629–0.2272)
Species estimation^a^						
*P. westermani*	38	1132	0.2464	0.2507 (0.2067–0.3102)	0.2255	0.2291 (0.1913–0.2801)
*P. skrjabini*	38	3459	0.1739	0.1770 (0.1496–0.2097)	0.1622	0.1650 (0.1408–0.1934)
Other	2	581	0.2073	0.2212 (0.1646–0.2983)	0.1951	0.2067 (0.1563–0.2729)
Unknown	3	130	0.1971	0.2014 (0.1537–0.2694)	0.1877	0.1912 (0.1468–0.2543)

^a^One article reported clinical symptoms of both *P. westermani* and *P. skrjabini* cases

*DW* Disability weight

Table 3 shows the DWs of the different types of paragonimiasis cases. The overall DW estimates of paragonimiasis were 0.1927 (using the additive approach) and 0.1791 (using the multiplicative approach), with 5302 clinical cases from 80 general articles. The DWs estimated using the multiplicative approach were generally lower than those estimated using the additive approach. The DWs of *P. westermani* cases were higher than those of *P. skrjabini* cases, in both reported and estimated species groups. However, DWs simulated with clinical cases of estimated species showed a smaller gap.

With 5000 Monte Carlo simulations, the findings of the uncertainty analysis showed the magnitude and robustness of DW estimates. The median value of DW estimates in the uncertainty analysis was 0.1956, with a 95% UI of 0.1632–0.2378 using the additive approach, while the value was 0.1816 with a 95% UI of 0.1530–0.2182 using the multiplicative approach (see Table 3).

Table 4 shows the results of a base case analysis of outcomes. Lung outcomes and headache were the two outcomes that were critical to the overall DW of paragonimiasis, with a greater than 10% change occurring when the specific outcome was missing from the overall disability weight estimation.

**Table 4:** Base case analysis of outcomes

Outcome	No. of cases with the outcome	Occurrence rate of cases with the outcome (%)	Overall DW % change without the outcome	
			Additive approach	Multiplicative approach
Lung outcomes	1425	26.88	38.91	37.16
Pleural outcomes	1341	25.29	7.09	6.35
Pericardial outcomes	271	5.11	6.68	5.98
Headache	614	11.58	26.50	24.67
Epilepsy	90	1.70	4.86	4.34
Motor loss	86	1.62	0.51	0.45
Vision impairment	23	0.43	0.07	0.06
Diarrhoea	268	5.05	1.94	1.72
Abdominal pain	879	16.58	5.16	4.60
Hepatomegaly	685	12.92	4.02	3.58
Skin rash	168	3.17	1.12	0.99
Subcutaneous mass	1391	26.24	3.13	2.78

*DW* Disability weight

The differences in DW estimates between various types of paragonimiasis were further analysed by comparing outcome contributions to the overall estimates.

**Fig. 2:**
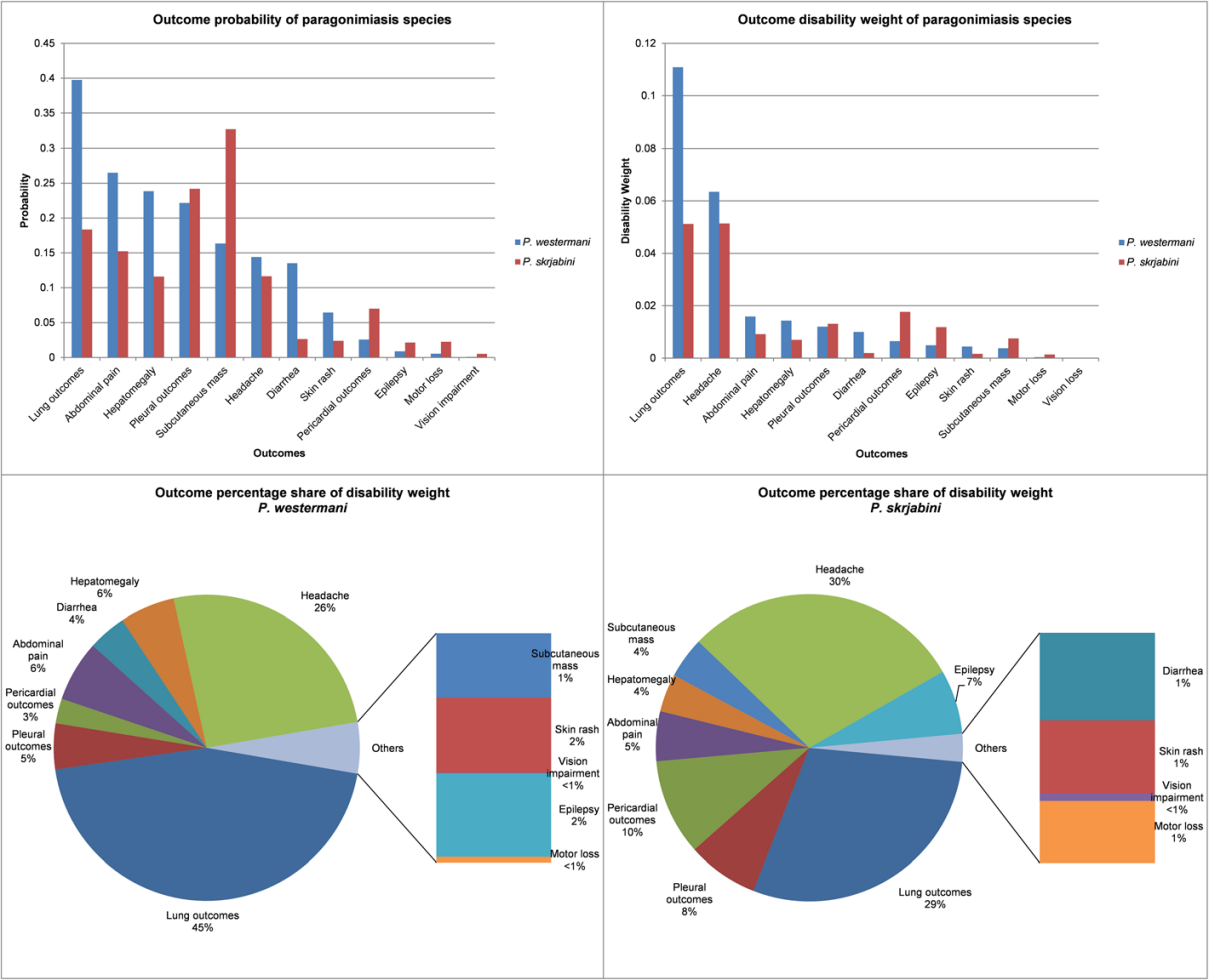
Outcome comparison by species. Figure 2 shows the comparison of the outcome probability, DW contribution, and its percentage share between *Paragonimus westermani* cases and *P. skrjabini* cases. DW: Disability weight

Figure 2 shows the comparison of the outcome probability, DW contribution, and its percentage share (using the additive approach) between *P. westermani* cases and *P. skrjabini* cases. Lung outcomes and headache were the top two contributors to DW estimates for both species. Compared to *P. skrjabini* cases, lung outcomes were more probable to occur in *P. westermani* cases and had more percentage share, thus bringing more contributions to the overall DW estimate of the latter species. Subcutaneous mass was more likely to occur in *P. skrjabini* cases, but resulted in less impact to its DW estimates compared with *P. westermani* cases (see Fig. 2).

## Discussion

Our study simulated the overall DW estimate of paragonimiasis at 0.1927 (median 0.1956) using the additive approach and 0.1791 (median 0.1816) using the multiplicative approach. The estimated DW of *P. westermani* was higher than that of *P. skrjabini.* Lung outcomes and headache contributed the greatest to DW estimates.

Compared with the GBD 2010 study, this study found considerably higher DWs than the health status of “moderate abdominal pelvic problem” (0.123) that Salomon et al. [15, 28] used as a proxy for the outcomes of both heavy paragonimiasis and cerebral paragonimiasis. However, abdominal pelvic problem is never the dominant manifestation even if sometimes abdominal organs could be involved [2].

Our DW estimates are also higher than those Fürst et al. [3] used for heavy paragonimiasis (0.099) and cerebral paragonimiasis (0.100). The reason could be that we included more outcomes in our model, such as headache and pericardial symptoms. The necessity of these extra outcomes was manifested by their non-negligible contribution to the overall DW. However, our DW estimates were lower than either the heavy paragonimiasis or cerebral paragonimiasis DWs used in GBD 2013 and GBD 2016 [16–18], as they focus on more severe situation with higher worm burden or cerebral involvement. The application of the two different types of DWs in the years lived with disability (YLD) calculation shall have to match with different incidence/prevalence rates. However, it was often difficult to find the morbidity information relating to heavy/cerebral paragonimiasis for a specific region in the literature search [18]. Therefore, the overall paragonimiasis DW estimated in this study that match just overall incidence/prevalence rate in YLD calculation could better fit the data paucity situation.

Our result is higher than the health status DW pertaining to acute episode of severe infectious disease (0.133) and a bit lower than that of post-acute effects (fatigue, emotional lability, and insomnia) of infectious diseases (0.219) as outlined in GBD 2013 [16, 24]. Compared with *Clonorchis sinensis*, another foodborne trematode disease, our estimate is higher than the DW simulated by Qian et al. [29] using data collected from a field survey. Compared with the DW Finkelstein et al. [26] estimated for schistosomiasis using a decision tree (0.132), our result is also higher. Apart from the difference between diseases, the reason could be partly due to data sources. Most of our recruited articles were case reviews based on clinical cases that referred to hospitals as field surveys were either unavailable or reported insufficient information. This may be associated with the overestimation of the severity of a disease.

We further found that headache contributed more to the DW estimation than epilepsy. Headache is probably a neglected outcome for cerebral paragonimiasis in disease burden estimation. None of the GBD studies [3, 15, 18, 30] considered headache as an important outcome of cerebral paragonimiasis. In a FERG estimation to compare DALYs of foodborne diseases, the DW of paragonimiasis also just considered the epilepsy outcome of cerebral symptoms [21]. Although paragonimiasis DALY ranked high in a comparison of foodborne diseases [22], it could still be underestimated with the neglect of headache impact to the DW used in calculation.

Our study generated DWs estimated using the additive approach and the multiplicative approach. The result showed that the DW estimated using the additive approach was generally higher than that estimated using the multiplicative approach. The limitation of the additive approach could be one of the reasons. Using this approach, the combined DW could exceed 1.0 and overestimation could occur when different outcomes affect the same health domain [27] (e.g. the lung outcome and the pericardial outcome could both lead to symptoms in the chest).

The DWs of *P. westermani* were higher than that of *P. skrjabini* for both reported and estimated species. For the reported species, their simulated 95% UIs did not overlap, while for the estimated species, their simulated 95% UIs overlapped for both the additive approach and the multiplicative approach. As the overlapped part was relatively small and the species was estimated based on the authors’ experiences, we still consider the difference to be somewhat significant. It was inferred that a possible reason could be the biological difference between the two species. *P. westermani* larvae develop into adult worms in the human lung, while only a few *P. skrjabini* larvae develop into adults in the same location. Most of the latter remain in the juvenile stage, migrating to different organs and causing damage [10]. Therefore, the analysis of outcome percentage share of the estimated DWs further showed that compared with *P. westermani*, the estimated *P. skrjabini* cases had higher percentage of extra-pulmonary outcomes.

In our study, only two articles with the species *P. africanus* and *P. miyazaki* were included in the modelling. The simulated DW was in between those of *P. westermani* and *P. skrjabini*. It was further inferred that some other species might be similar to *P. westermani* in DW estimates as they mainly cause pulmonary symptoms, including *P. africanus*, *P. heterotremus*, *P. uterobilateralis*, *P. kellicotti*, and *P. mexicanus*. These species distribute across West Africa, South and Southeast Asia, and North and South America [2]. Some other species could be similar to *P. skrjabini* with ectopic forms more common, such as *P. miyazaki* from Japan, which is very close morphologically and molecularly to *P. skrjabini* from China [2].

We recognize that our study had some limitations. We excluded case reviews in languages other than Chinese and English due to article availability and language barriers. We missed articles with full text non-available to us. This may bring bias to our study, however, we tried to minimize impact as most of the missed 32 articles were published earlier than in 1995. We also omitted some scarce outcomes such as spinal symptoms and eye symptoms, which may lead to an underestimation of the overall DW. In the uncertainty analysis, we replaced the missing range of some outcome DWs with the average value of available uncertainty levels. This may result in our uncertainty results having lower credibility. In addition, as most Chinese cases did not report species information, we estimated the species based on authors’ experiences on species information collected from cases in the same location. This kind of estimation may reduce the solidity of species classification.

Despite these potential limitations, our estimation of the DW of paragonimiasis helps to close the gap in the current disease burden system, particularly for NTDs. In low- and middle-income countries, such as China, paragonimiasis is increasingly becoming a potential threat, as the production and consumption of crayfish, the second intermediate host, has greatly risen in recent years. The national production of crayfish increased by 36.59% in 2017 compared with 2016, and for some provinces, the rate was as high as 141.05%; meanwhile, the number of crayfish-specific restaurants boomed in major cities like Shanghai and Guangzhou [31]. We call for more attention on this chronic disabling and death threatening “poverty disease” [32]. More studies are needed in areas related to paragonimiasis assessment, prevention, and treatment.

## Conclusions

Our study systematically estimated the DWs of paragonimiasis from reported cases for the first time. *P. westermani* was found to have higher DW estimates than *P. skrjabini*. These could bring considerable changes to disease burden estimation, thus having an impact on health policymaking and resource allocation in research, monitoring, and control, particularly when faced with the potentially increasing threat of paragonimiasis in low- and middle-income countries.

## Additional file

Additional file 1: Multilingual abstracts in the five official working languages of the United Nations. (PDF 225 kb)

Additional file 2: Data modelling details. (DOCX 26 kb)

## Abbreviations

COPD: Chronic obstructive pulmonary disease
DALY: Disability-adjusted life year
DW: Disability weight
FERG: Foodborne Disease Burden Epidemiology Reference Group
GBD: Global burden of disease
HIV: Human immunodeficiency virus
NTD: Neglected tropical disease
TB: Tuberculosis
UI: Uncertainty interval
WHO: World Health Organization
YLD: Years lived with disability
