# Supplementary material for: Estimation of disability weight for paragonimiasis: a systematic analysis
Source: Infect Dis Poverty. 2018 Oct 19;7:110. doi: 10.1186/s40249-018-0485-5 (PMC6196032; doi:10.1186/s40249-018-0485-5)
Supplement: Supplementary file 1 — Multilingual abstracts in the five official working languages of the United Nations. (PDF 225 kb) [file 40249_2018_485_MOESM1_ESM.pdf]

Translation of the abstract into the five official working languages of the United Nations

تقدير عبء العجز لداء جانبية المناسل: تحليل منهجي

يون فينج ، توماس فيرست ، لو لي و جو-جينج يانج

الملخص
الخلفية: داء جانبية المناسل ، الناجم عن الديدان الطفيلية من جنسالديدان المثقوبة الرئوية ، هو مرض استوائي مهمل. معاناة الإنسان من داء جانبية المناسل غالباً ما يساء فهمها ويختلف تقديرها إلى حد كبيربحسب عبء العجز في سنوات العمر المصححة باحتساب مدة العجز في تقديرات العبء العالمي للأمراض (GBD) المختلفة. هذا البحث هو لمراجعة الحالات المرضية السريرية لداء جانبية المناسل بشكل منهجي ولإعادة تقييم عبء العجز لداء جانبية المناسل البشري.
الطرق: تم إجراء تحليل منهجي باستخدام مقالات مأخوذة من قواعد البيانات التالية: PubMed ، ومعهد شبكة المعلومات العلمية للعلوم ، والبيانات الأساسية للمعرفة الوطنية بالصين، وقواعد بيانات المجلة العلمية الصينية Wanfang Data و CQVIP ، و Africa Journal Online ، ونظام المعلومات عن المطبوعات غير الرسمية في أوروبا. مصطلحات البحث هي مزيج من "paragonim*" مع "السريرية" أو "العدوى". تم تعيين فقط المقالات التي تحقق الشروط التالية لهذه الدراسة: تم الإبلاغ عن حدوث علامات وأعراض سريرية لداء جانبية المناسل في البشر ؛ تم تأكيد التشخيص، لم يتم الإبلاغ عن أي أمراض مصاحبة ؛ الحالات السريرية المراجعة أو النتائج الوبائية لم تكن مدرجة بالفعل في أي مقالات أخرى. تم استخراج المعلومات وتكرار نتائج جانبية المناسل من المقالات المشمولة باستخدام حقول بيانات محددة مسبقًا مرتين من قبل شخصين منفصلين. تم اختيار أعباء العجز بشكل رئيسي من مجموعات بيانات العبء العالمي للمرض GBD لعام 2004 و 2013. تم وضع نماذج لأعباء التكرار والعجز لداء جانبية المناسل في شجرة القرار باستخدام النهج الإضافي والنهج المضاعف ، على التوالي. تم تشغيل محاكاة مونت كارلو 5000 مرة لتحليل عدم القطعية.
النتائج: تم محاكاة تقديرات عبء العجز من داء جانبية المناسل عند 5302 حالات سريرية من 80 مقالة عامة. تم تقدير عبء العجز الكلي عند 0.1927 (متوسط 0.1956) مع فاصل عدم قطعية بنسبة 95٪ (UI) من 0.1632-0.2378 باستخدام النهج الإضافي ، و 0.1791 (متوسط 0.1816) مع فاصل عدم قطعية بنسبة 95٪ من 0.1530-0.2182 باستخدام النهج المضاعف. كانت أعباء العجز المحاكة لـ حالات جانبية المناسل الفسترمانية أعلى من حالات المثقوبة الرئوية. وكانت نتائج الرئة والصداع أكبر اثنين من المساهمين في عبء العجز لكلا النوعين.
الاستنتاجات: يجب إعادة النظر في استخدام عبء داء جانبية المناسل فيما يتعلق بتوافر بيانات المرض وتغير الأنواع. يتطلب حساب عبء المرض لداء جانبية المناسل مزيدًا من التعديل ومن ثم يكون له آثار كبيرة على أولويات الصحة العامة في البحث والمراقبة والتحكم.

Translated from English version into Arabic by Free bird and Mohannad Aljarrah, through

TRANSLATORS
WITHOUT BORDERS

肺吸虫病伤残指数评估：一项系统分析

冯云，Thomas Fürst，刘璐，杨国静

摘要
引言：由并殖吸虫属蠕虫引起的肺吸虫病是一种被忽视热带病。肺吸虫病对人类造成的病痛经常被误解，并且不同全球疾病负担（GBD）评估体系中伤残调整寿命年的伤残指数对其的定量各有不同。本文系统回顾了临床肺吸虫病例并重新定量评估人类肺吸虫病伤残指数。
方法：通过利用以下数据库来源的文章进行系统分析：PubMed, Institute for Scientific Information Web of Science，中国知网，中国科学期刊数据库万方及重庆维普，Africa Journal Online, and the System for Information on Grey Literature in Europe. 搜索条目为“paragonim*”，

"clinical"，"infection"的联合。只有满足以下条件的文章才能入选本研究：报道了人类肺吸虫病临床表现和症状的发生率；确诊病例；没有合并其他疾病；其他文章没有重复报道过入选的临床病例或流行病研究。两个研究人员用事先设计好的数据表格分别从入选文章中提取肺吸虫病临床后果的信息和发生频率两遍。临床后果的伤残指数主要从GBD 2004和GBD 2013数据库中选取。在决策树模型中，用附加法和乘性法分别处理肺吸虫病临床后果的发生频率和伤残指数。再用蒙特卡洛模拟法运行 5,000 次进行不确定分析。
结果：通过 80 篇全面报道的文章中的 5,302 例临床病例对肺吸虫病伤残指数进行估计。附加法得到总伤残指数为 0.1927 （中位数 0.1956， 95%不确定区间 0.1632-0.2378），乘性法得到总伤残指数为 0.1791 （中位数 0.1816， 95%不确定区间 0.1530-0.2182）。计算得到的卫氏肺吸虫病伤残指数高于斯氏肺吸虫病。在两个虫种中，肺部临床后果和头痛表现均位列伤残指数贡献前二位。
结论：肺吸虫病伤残指数的应用需要根据发病数据可及性及虫种问题来重新评估。肺吸虫疾病负担的计算需要进一步的修正，因而会对公共卫生研究、监控和管理的优先安排产生相当大的影响。

Translated from English version into Chinese by Yun Feng

Estimation du poids de l'incapacité lié à la paragonimiase : une analyse systématique

Yun Feng, Thomas Fürst, Lu Liu et Guo-Jing Yang

Résumé

Contexte : La paragonimiase, causée par des helminthes du genre *Paragonimus* spp., est une maladie tropicale négligée. Les personnes souffrant de paragonimose sont souvent mal comprises et la quantification de la maladie par le poids de l'incapacité, utilisé pour le calcul de la mesure d'années de vie corrigées du facteur invalidité, varie grandement en fonction des différentes estimations de la charge mondiale de morbidité. Le présent document a pour but d'examiner systématiquement les cas cliniques de paragonimiase, et de requantifier le poids de l'incapacité lié à la paragonimiase humaine.
Procédés: Une analyse systématique a été menée sur la base d'articles tirés des bases de données suivantes : PubMed, l'Institute for Scientific Information, le Web of Science, CNKI (China National Knowledge Infrastructure), la base de données du Chinese scientific journal, Wanfang Data et CQVIP, l'Africa Journal Online et le Système d'information sur la littérature grise en Europe. Pour la recherche, on a utilisé une combinaison de « paragonim* » avec « clinique » ou « infection ». Seuls les articles remplissant les conditions suivantes ont été retenus pour la présente étude : l'apparition de signes et symptômes cliniques de paragonimiase chez les êtres humains ont été signalés ; le diagnostic a été confirmé ; aucune comorbidité n'a été signalée ; les cas cliniques ou les résultats épidémiologiques examinés n'ont pas déjà été inclus dans d'autres articles. L'information et la fréquence des résultats liés à la paragonimiase des articles inclus utilisant des champs de données prédéfinis ont été extraites deux fois par deux individus distincts. Les résultats (poids de l'incapacité) ont été sélectionnés principalement à partir des ensembles de données GBD 2004 et GBD 2013. Les fréquences et les poids d'incapacité des résultats liés à la paragonimiase ont été modélisés sous forme d'un arbre de décision en utilisant respectivement l'approche additive et l'approche multiplicative. Des simulations à l'aide de la méthode de Monte-Carlo ont été effectuées 5000 fois pour une analyse d'incertitude.
Résultats : Les estimations du poids de l'incapacité de la paragonimiase ont été simulées avec 5302 cas cliniques, tirés de 80 articles généraux. Le poids global de l'incapacité a été estimé à 0,1927

(médiane 0,1956) avec un intervalle d'incertitude (IU) de 95% de 0,1632-0,2378 en utilisant l'approche additive, et 0,1791 (médiane 0,1816) avec un IU de 95% de 0,1530-0,2182 en utilisant l'approche multiplicative. Les poids d'invalidité simulés des cas *Paragonimus westermani* étaient plus élevés que ceux des cas de *P. skrjabini*. Les résultats pulmonaires et les maux de tête ont été les deux principaux facteurs ayant contribué au poids de l'incapacité pour les deux espèces.
Conclusions : Il faudra reconsidérer l'utilisation du poids de l'incapacité lié à la paragonimiase en tenant compte de la disponibilité des données de morbidité et de la variation des espèces. Le calcul de la charge de morbidité de la paragonimiase nécessite d'autres modifications et a donc des conséquences notables pour l'établissement des priorités de santé publique en matière de recherche, de surveillance et de contrôle.

Translated from English version into French by Ghizlane J and Alice Montergnole, through

TRANSLATORS WITHOUT BORDERS

Оценка весового коэффициента инвалидности для парагонимоза: систематический анализ

Юнь Фэн, Томас Фюрст, Лу Лю и Го-Цзин Ян

Аннотация

Краткое описание: Парагонимоз, возбудителем которого служат гельминты рода *Paragonimus* spp., является забытой тропической болезнью. Страдания людей от парагонимоза часто неверно истолкованы, а его квантификация по весовому коэффициенту инвалидности при прогнозе продолжительности жизни с поправкой на нетрудоспособность существенно варьируется при разных оценках глобального бремени болезни (GBD). В данной статье проведён систематический обзор клинических случаев заболевания парагонимозом и сделана повторная квантификация весового коэффициента инвалидности, вызванной парагонимозом человека.
Методы: Систематический анализ был выполнен с использованием статей из следующих баз данных: PubMed, поисковой платформы Web of Science Института научной информации, Китайской национальной инфраструктуры знаний (CNKI), китайских научных журналов Wanfang Data и CQVIP, электронной библиотеки научных публикаций Африки Africa Journal Online, а также системы информации по внеиздательской литературе в Европе. Поисковые термины представляли собой комбинацию понятия «парагоним*» со словами «клинический» или «инфекция». Для настоящего исследования были отобраны только статьи, удовлетворявшие следующим условиям: у людей было зарегистрировано наличие клинических признаков и симптомов парагонимоза; диагноз был подтверждён; отсутствовали сообщения о сопутствующих болезнях; рассмотренные клинические случаи или эпидемиологические заключения на указанный момент времени не были включены в другие статьи. Из включённых статей с помощью предопределённых полей данных два раза и двумя разными сотрудниками были выделены информация, а также частота осложнений при заболевании парагонимозом. Весовые коэффициенты инвалидности при осложнениях в результате заболевания были в основном выбраны из наборов данных GBD 2004 и GBD 2013. Моделирование частоты и весовых коэффициентов инвалидности при осложнениях, вызванных парагонимозом, было выполнено в виде дерева решений с использованием

соответственно аддитивного и мультипликативного подходов. Для анализа неопределённости было выполнено 5000-кратное имитационное моделирование методом Монте-Карло.
Результаты: Оценки весовых коэффициентов инвалидности при заболевании парагонимозом были смоделированы по 5302 клиническим случаям из 80 общих статей. По проведённым оценкам, общий весовой коэффициент инвалидности составил 0,1927 (медианное значение 0,1956) с интервалом неопределенности (UI) 95% в диапазоне 0,1632–0,2378 при использовании аддитивного подхода и 0,1791 (медианное значение 0,1816) с интервалом неопределенности (UI) 95% в диапазоне 0,1530–0,2182 при использовании мультипликативного подхода. Смоделированные весовые коэффициенты инвалидности по случаям заболевания *Paragonimus westermani* были выше аналогичных коэффициентов по случаям заболевания *P. skrjabini* . В обоих случаях двумя главными факторами, повлиявшими на весовые коэффициенты инвалидности, стали осложнения на лёгкие и головная боль.
Выводы: Необходимо пересмотреть применение весового коэффициента инвалидности по парагонимозу с учётом доступности данных о заболеваемости и изменчивости видов. Вычисление бремени парагонимоза требует дальнейшей модификации и тем самым значительно влияет на приоритизацию в области общественного здравоохранения при проведении исследований, мониторинга и контроля.

Translated from English version into Russian by Anna Romanenko and Liudmila Tomanek, through

TRANSLATORS
WITHOUT BORDERS

Estimación del grado de discapacidad para la paragonimiasis: un análisis sistemático

Yun Feng, Thomas Fürst, Lu Liu y Guo-Jing Yang

Resumen

Escenario La paragonimiasis, causada por los helmintos del tipo *Paragonimus*es una enfermedad tropical desatendida. Los humanos que padecen paragonimiasis a menudo no son tenidos en consideración y su cuantificación según el grado de la discapacidad de los años de vida de discapacidad ajustada varían en gran medida para las diferentes estimaciones de los grados de la enfermedad (GBD). Este informe sirve para revisar sistemáticamente los casos de paragonimiasis clínica y recuantificar el grado de discapacidad de la paragonimiasis humana
Métodos Se llevó a cabo un análisis sistemático usando artículos de las siguientes bases de datos: PubMed, Institute for Scientific Information Web of Science, China National Knowledge Infrastructure, las bases de datos del Chinese scientific journal Wanfang Data y CQVIP, Africa Journal Online, y el System for Information on Grey Literature de Europa. Los términos de búsqueda eran la combinación de "paragonim*" con "clínico" o "infección". Para este estudio solo se tuvieron en cuenta los artículos que cumplieran con las siguientes condiciones: se había informado de la aparición de señales y síntomas clínicos de la paragonimiasis en humanos; se había confirmado el diagnóstico; no se había informado de comorbilidades; los casos o hallazgos epidemiológicos revisados no habían sido incluidos todavía en ningún otro artículo. La información y frecuencias de los resultados de la paragonimiasis a partir artículos incluidos que usaban campos de datos predefinidos fueron extraídos dos veces por dos personas diferentes. Los resultados de los grados de discapacidad fueron seleccionados en su mayoría de los datos de los GBD 2004 y GBD

2013. La información y frecuencias de los resultados de la paragonimiasis fueron transformados en un árbol de decisiones usando el acercamiento aditivo y multiplicativo, respectivamente. Se realizaron 5000 simulaciones de Monte Carlo para los análisis inciertos.

Resultados: Los grados estimados de discapacidad de la paragonimiasis se simularon en 5302 casos clínicos de 80 artículos generales. El grado general de discapacidad se estimó en 0,1927 (promedio 0,1956) con un 95 % de intervalo de incertidumbre (IU) de 0,1632–0,2378 usando el acercamiento aditivo, y 0,1791 (promedio 0,1816) con un 95 % de IU de 0,1530–0,2182 usando el acercamiento multiplicativo. Los grados de discapacidad simulada de los casos de *Paragonimus westermani* eran mayores que los de los casos de *P. skrjabini*. Las afecciones de pulmón y el dolor de cabeza eran los dos mayores contribuidores al grado de discapacidad para ambas especies.

Conclusiones El uso del grado de discapacidad de la paragonimiasis debe reconsiderarse en lo que a disponibilidad de morbilidad y variación de la especie se refiere. Al calcular el grado de la enfermedad de la paragonimiasis es preciso avanzar con las modificaciones y ello tiene implicaciones para la sanidad pública en la prioridad de investigación, monitorización y control.

Translated from English version into Spanish by Raquel Hurtado and Constanza Olivares R, through

TRANSLATORS
WITHOUT BORDERS
