# Supplementary material for: Estimation of disability weight for paragonimiasis: a systematic analysis
Source: Infect Dis Poverty. 2018 Oct 19;7:110. doi: 10.1186/s40249-018-0485-5 (PMC6196032; doi:10.1186/s40249-018-0485-5)
Supplement: Supplementary file 2 — Data modelling details. (DOCX 26 kb) [file 40249_2018_485_MOESM2_ESM.docx]

**Decision Tree Modeling**

The decision tree model was used to combine different outcomes together. As the twelve outcomes did not have a sequential order. Therefore, they were at the parallel branch of the tree with the decision yes or no to be made. Two approaches were used to calculate the DW of paragonimiasis: the additive approach and the multiplicative approach with the following formulas:

Formula 1 (Additive approach):

$$DW=\sum_{A=1}^{n} P_{outcome A}*{DW}_{outcome A}$$

Formula 2 (Multiplicative approach):

$$DW=1-\sum_{A=1}^{n} {(1-P}_{outcome A}*{DW}_{outcome A})$$

DW_outcome A_ represents the disability weight of outcome A. P_outcome A_ represents the probability/frequency of outcome A. All together twelve outcomes were included (see Table 2 in the article). P_outcome A_ was calculated by the total number of cases with outcome A divided by the total number of included cases in the group. The total number of cases with different outcomes was displayed in the following table:

<Table 1 inserted here>

**Table 1. Number of cases with different outcomes in different groups**

| **Outcomes** | **Total No.** | **Species Reported** | | **Species Estimation** | |
| --- | --- | --- | --- | --- | --- |
|  |  | ***P.westermani*** | ***P.skrjabini*** | ***P.westermani*** | ***P.skrjabini*** |
| Lung outcome | 1425 | 128 | 113 | 450 | 635 |
| Pleural outcome | 1341 | 70 | 199 | 251 | 837 |
| Pericardial outcome | 271 | 2 | 10 | 29 | 242 |
| Headache | 614 | 88 | 8 | 163 | 403 |
| Epilepsy | 90 | 2 | 5 | 10 | 74 |
| Motor loss | 86 | 1 | 1 | 6 | 78 |
| Vision impairment | 23 | 0 | 0 | 1 | 18 |
| Diarrhea | 268 | 1 | 13 | 153 | 91 |
| Abdominal pain | 879 | 4 | 3 | 300 | 527 |
| Hepatomegaly | 685 | 20 | 3 | 270 | 401 |
| Skin rash | 168 | 0 | 0 | 73 | 83 |
| Subcutaneous mass | 1391 | 12 | 238 | 185 | 1133 |

**Base Case Analysis**

The purpose of base case analysis was to understand the composition of DW estimates and to find out the critical outcomes that contributed the most. Therefore the percentage changes were calculated and compared for both additive approach and multiplicative approach supposing the specific outcome was missing.

Formula 3.

$$Percentage Change= \frac{(DW- {DW}_{no outcome A})}{DW}\times100\%$$

DW represents the original DW with all outcomes included. DW _no outcome A_ represents the DW calculated without the contribution of outcome A.

**Uncertainty Analysis**

The uncertainty analysis used the method of probabilistic sensitivity analysis. In formula 1 & 2, the DW estimates depended on the value of P_outcome A_ and DW_outcome A_. We assumed the P_outcome A_ followed the beta distribution, which distributed within 0 and 1. The parameters were estimated using formula 4 & 5.

Formula 4.

$$a={SUM}_{outcome A}+1$$

Formula 5.

$$b= SUM+1-{SUM}_{outcome A}$$

SUM_outcome A_ represents the total case number with the outcome A. SUM represents the total case number included in the group.

DW_outcome A_ followed the lognormal distribution. Log-means and log-SDs were estimated by the 2.5% and 97.5% value reported (Table 2).

<Table 2 inserted here>

**Table 2. Lognormal distribution of outcome DWs**

| **Outcome** | **Mean** | | **2.5%** | | **7.5%** | | **Log-mean** | | **Log-SD** |
| --- | --- | --- | --- | --- | --- | --- | --- | --- | --- |
| **Pleural outcome** | 0.054 | 0.035 | | 0.079 | | -2.91877 | | 0.207679 | |
| **Diarrhea** | 0.074 | 0.049 | | 0.104 | | -2.60369 | | 0.191982 | |
| **Headache** | 0.441 | 0.294 | | 0.588 | | -0.81871 | | 0.176823 | |
| **Epilepsy** | 0.552 | 0.375 | | 0.71 | | -0.59421 | | 0.162842 | |
| **Motor Loss** | 0.061 | 0.04 | | 0.089 | | -2.79688 | | 0.20402 | |
| **Vision Impairment** | 0.031 | 0.019 | | 0.049 | | -3.47377 | | 0.241679 | |
| **Pericardial outcome** | 0.252 | -- | | -- | | -1.37833 | | 0.197504* | |
| **Lung Outcome** | 0.279 | -- | | -- | | -1.27654 | | 0.197504* | |
| **Abdominal Pain** | 0.06 | -- | | -- | | -2.81341 | | 0.197504* | |
| **Hepatomegaly** | 0.06 | -- | | -- | | -2.81341 | | 0.197504* | |
| **Skin Rash** | 0.068 | -- | | -- | | -2.68825 | | 0.197504* | |
| **Subcutaneous Mass** | 0.023 | -- | | -- | | -3.77226 | | 0.197504* | |

*The log-SDs for the outcome without 2.5% and 97.5% values were replaced by the mean values of the rest of outcomes.

Values were randomly generated for DW_outcome A_ and P_outcome A_ from their distributions respectively. And DW estimates were then calculated using both additive approach and multiplicative approach. The whole process was run for 5000 times to generate the distribution of DW estimates. The 2.5% and 97.5% values were used to represent the 95% uncertainty interval.
